# Transplantation of endothelial progenitor cells in treating rats with IgA nephropathy

**DOI:** 10.1186/1471-2369-15-110

**Published:** 2014-07-09

**Authors:** Wei Guo, Jiang-Min Feng, Li Yao, Li Sun, Guang-Qing Zhu

**Affiliations:** 1 Department of Nephrology, The Fourth Hospital of People, Shenyang 110020, China; 2 Department of Nephrology, The First Affiliated Hospital of China Medical University, Shenyang 110001, China; 3 Department of Meical Ultrasound, The Fourth Hospital of People, Shenyang 110020, China

**Keywords:** Endothelial progenitor cells, IgA nephropathy, Peritubular capillary, Transplantation

## Abstract

**Background:**

Therapeutic options in IgAN are still limited. The aim of this study is to explore the feasibility of using endothelial progenitor cell to treat IgAN in rat model.

**Methods:**

Rat bone marrow mononuclear cells (BM-MNCs) obtained with density gradient centrifugation were cultured *in vitro*, and induced into endothelial progenitor cells (EPCs). EPCs were identified by surface marker CD34, CD133 and VEGFR2 (FLK-1) and by Dil-Ac-LDL/FITC-UEA-1 double staining. EPCs were labeled with PKH26 prior to transplantation. Rat model of IgAN was established by oral administration of bovine serum albumin together with lipopolysaccharide via the caudal vein and subcutaneous injection of CCL_4_. Kidney paraffin sections were stained by H&E and PAS. Immunofluorescence was used to assess IgA deposition in the glomeruli. Peritubular capillary (PTC) density was determined by CD31 staining. Monocyte chemoattrant protein-1 (MCP-1), hypoxia-inducible factor-1α (HIF-1α) and CD105 were also measured by immunohistochemistry, western blotting and real-time fluorescent quantitative PCR.

**Results:**

The transplanted BM-EPCs were successfully located in IgAN rat kidney. After transplantation, Urinary red blood cell, urine protein, BUN, Scr and IgA serum level were significantly decreased, so were the areas of glomerular extracellular matrix and the IgA deposition in the glomeruli. In addition, PTC density was elevated. And the expression levels of HIF-1α and MCP-1 were significantly down-regulated, while the expression of CD105 was up-regulated. All these changes were not observed in control groups.

**Conclusion:**

The BM-EPCs transplantation significantly decreases the expansion of glomerular extracellular matrix and the deposition of IgA in the glomeruli; lowers the expression of inflammatory factors; increases PTC density; improves ischemic-induced renal tissue hypoxia, all of which improves the renal function and slows the progress of IgA nephropathy.

## Background

IgAN, the most common form of glomerular disease throughout the world, was first reported by Berger and Hinglais in France in 1968 [1]. 25% patients with IgAN will develop end stage renal diseases (ESRD), 5–25 years after the diagnosis of IgAN. However, the cause of primary IgAN and the mechanism defining mesangial IgA deposition in IgAN are unclear, and thus there has been no effective therapeutic treatment for patients with IgAN so far.

Glomerulosclerosis and interstitial fibrosis are irreversible pathological changes during the development of IgAN to ESRD. Therefore, the focus of our research on how to delay the progress of renal fibrosis in IgAN patients. Previous studies have shown that in IgAN patients, cell damage and apoptosis of renal microvascular endothelial cells and loss of PTC cause renal ischemia, hypoxia, activation of stromal cell, secretion of fibrosis cytokines, which leads to the upregulation of fibrosis-related genes and accumulation of extracellular matrix (ECM) [2-5]. In 1997, Asahara et al. [6], for the first time, isolated and confirmed the existence of endothelial progenitor cells, which can differentiate into vascular endothelial cells and can be used for angiogenesis *in vivo*. Currently, most studies suggest that the EPC is mainly derived from umbilical cord blood, adult bone marrow and peripheral blood [7]. Therefore, the aim of our study is to investigate whether BM-EPCs transplantation will be an effective therapeutic mean for IgAN patients.

## Methods

### Animal

The China medical university animal care and use Committee approved the procedures.

Female Sprague–Dawley (SD) rats, weighted 150–200 gram, were used in this study. The rats were maintained under a 12 hour light/dark cycle at a constant temperature (22 ± 1°C), with free access to clean water and normal chow. The rats were orally administrated with BSA (MBCHEM, USA) at dosage of 400 mg/kg, continuously for 6 weeks. At the 6.8^th^ week, the rats received lipopolysaccharide (LPS) (AMRESCO Inc, Solon, OH, USA) injection via the caudal vein, at a dose of 0.25 mg/kg. Then, the rats were received subcutaneous injection of 0.4 ml castor oil and 0.1 ml carbon tetrachloride (CCL_4_) once a week, continuously for 9 weeks as described before, with slight modification [8-10]. To minimize the effect of CCL_4_ on liver, the dosage of CCL_4_ was reduced to one third of the dosage used for establishment of hepatic fibrosis

The IgAN rats were identified by measuring 24-hour urine protein, Urinary red blood cell, blood urea nitrogen (BUN), serum creatinine (Scr), as well as level of IgA. Meanwhile, Liver function was monitored by Alanine amino transferase (ALT), aspertate amino- transferase (AST) and γ-glutamyl- transferase (GGT) were tested. Each kidney was divided into 2 parts. The first part was embedded in paraffin and stained with hematoxylin and eosin (H&E). The second portion was embedded in OCT and stored at −70°C followed by frozen sectioning for immunofluorescence staining. Direct immunofluorescence (FITC-conjugated rabbit anti-rats IgA antibody from Dako, USA) was used to visualize the IgA deposition in the glomeruli. The IgA deposition was graded by a 5-stage semi-quantitative method (− to++++): (−), no staining under low magnification and possible staining under high magnification; (+), possible staining under low magnification and staining under high magnification; (++), staining under low magnification and clear staining under high magnification; (+++) clear staining under low magnification and bright staining under high magnification; (++++), very intense staining under high magnification. Then, the IgAN model rats were randomly divided into two groups: IgAN group (IgAN + saline group, 6 rats) and EPC group (IgAN + endothelial progenitor cells, 30 rats). Normal rats receiving saline (n = 6) were used as control group. The study was approved by the Academic Review Board at China Medical University (see Additional file 1).

### Culture and identification of EPCs

EPCs were isolated and prepared by using the density gradient method, according to the previous description [6,11,12]. The femur and tibia of SD rats were separated under sterile conditions, from which bone marrow mononuclear cells were isolated. At 37°C 5% CO_2_ incubator, the cells were seeded in 6-well plates, at 5 × 10^5^ cells/well, in M199 medium supplied with VEGF 10μg/L, β-FGF 5μg/L, EGF 5μg/L, IGF5μg/L, 10% fetal bovine serum (HyClone Inc., Logan, Utah, USA). At day 14, the cells became spindle- shaped. The expression of CD34 (Santa Cruz Biotechnology, Inc, Santa Cruz, CA, USA), CD133 (Abnova Inc., Taipei, China) and VEGFR2 (Peprotech, Rocky Hill, NJ, USA) were identified by flow cytometer (BD Inc., San Jose, CA, USA). The EPCs were selected by Dil-ac-LDL/FITC-UEA-1(Molecular Probes, Eugene, OR) dual staining, and were labeled by PKH26 before prior to transplantation.

### Transplantation of EPCs

EPCs were injected 3 days after the induction of lgAN. EPC group rats received 3 × 10^6^ cells per rat via the caudal vein (0.5 ml/rat). Other rats received same volume of 0.9% saline. Six rats in EPC group were sacrificed at 1, 3, 7, 14 days after transplantation and 1 day before transplantation. Rats of other groups were sacrificed at 14 days after transplantation. 24 hour urine protein, Urinary red blood cell, BUN and Scr were examined for all rats. Each kidney was divided into three parts: one for pathological analysis and immunohistochemistry studies; The second portion was embedded in OCT for immunofluorescence staining; The third part was saved in liquid nitrogen for western blotting and real time RT-PCR.

### Identify the renal tissue morphology and histochemistry studies

The renal tissues were embedded in paraffin. The sections were cut at 3–4 μm thickness, and stained with H&E and PAS. Five pictures were taken randomly for each PAS-stained section at 400× magnification, and were analyzed by MetaMorph software (UIC) to calculate the ratio of glomerular ECM area and total glomerular area, which was used as the thickness index of glomerular mesangial matrix. Immunohistochemical staining on renal section was performed with ABC method. Polyclonal antibodies against CD31, HIF-1α, MCP-1 and CD105 were purchased from Bioss Inc. (Beijing, China). Five pictures were randomly taken for each section at 400× magnification, and integral optical density (IOD) was calculated by microscopic image analyzer (MetaMorph/DP10/BX41, UIC/Olympus, USA/Japan). IOD value of CD31 (endothelial cell-specific marker) was used to represent the PTC density.

### Western blotting assay

The total protein was extracted from renal tissue by RIPA lysis buffer (Beyotime Inc., Shanghai, China), and separated by 10% SDS-PAGE. Then, the separated protein was transferred to nitrocellulose membrane (70 V for 1.5 hours). The blots were blocked in 5% non-fat milk for 1 hour at room temperature, and then incubated with primary antibodies (HIF-1α, 1:500; MCP-1, 1:500; CD105, 1:400) overnight at 4°C. At the second day, horseradish peroxidase labeled secondary antibody (goat anti-rabbit IgG- HRP, 1:1000) were incubated with the blots at room temperature for 2 hours. Then, the blots were washed and incubated with NBT/BCIP for 10–30 mins. The blots were then scanned and analyzed by Gel-Pro Analyzer software (Media Cybernetics, Silver Spring, MD). The relative expression levels were quantified and normalized to control groups.

### Real-time fluorescent quantitative RT-PCR

Total RNA extracted by TRIzol extraction (Tiangen Inc., Nanjing, China) of kidney homogenates, and quantified by UV spectrophotometer. Purity of RNA was identified by A_260_/A_280_ ratio and gel electrophoresis. Primers for CD105, MCP-1, HIF-1α and β-actin (internal control) were designed with Primer Premier 5.0 software (Palo Alto CA, USA) as shown in Table 1. 2 μg of total RNA were reverse-transcribed into cDNA by using TIANScriptcDNA Kits (Tiangen Inc., Nanjing, China). Real-time quantitative PCR were then performed with SYBR Premix Ex Taq (Clontech, Mountain View, CA) in 20 μl volume, in the following condition: 95°C × 5 s, 60°C × 20s, 72°C × 30 s, for 40 cycles. Each sample was measured three times, and the average of Ct was taken. The relative expressions of mRNA were measured using 2^−ΔΔCt^ method.

**Table 1:** Sequences of amplification primers for CD105, MCP-1, HIF-1 and β-actin

**Name**	**Sequence (5′¬3′)**	**Tm (°C)**	**Size (bp)**
CD105 F	ACTCGGGAGGTGTTTCTGGTCTT	63.1	211
CD105 R	GTGCTGCTATGGAGGTAATGGTG	61.4	
MCP-1 F	TGAGTCGGCTGGAGAACTACAAG	61.3	209
MCP-1 R	AGGTGCTGAAGTCCTTAGGGTTG	61.7	
HIF1-αF	GCCTTAACCTATCTGTCACTTTG	55.9	250
HIF1-αR	ATTGTCTTCTGCTCCATTCCAT	58	
β-actin F	GGAGATTACTGCCCTGGCTCCTAGC	60.1	155
β-actin R	GGCCGGACTCATCGTACTCCTGCTT	62	

### Statistical analysis

Data was analyzed with one-way ANOVA analysis of variance using SPSS software 17.0 (IBM, Armonk, NY, USA). Data were presented as the mean ± S.E.M of at least three measurements. *P <* 0.05 was considered as significant in comparison with control.

## Results

### Characterization of IgAN animal model

**Figure 1:**
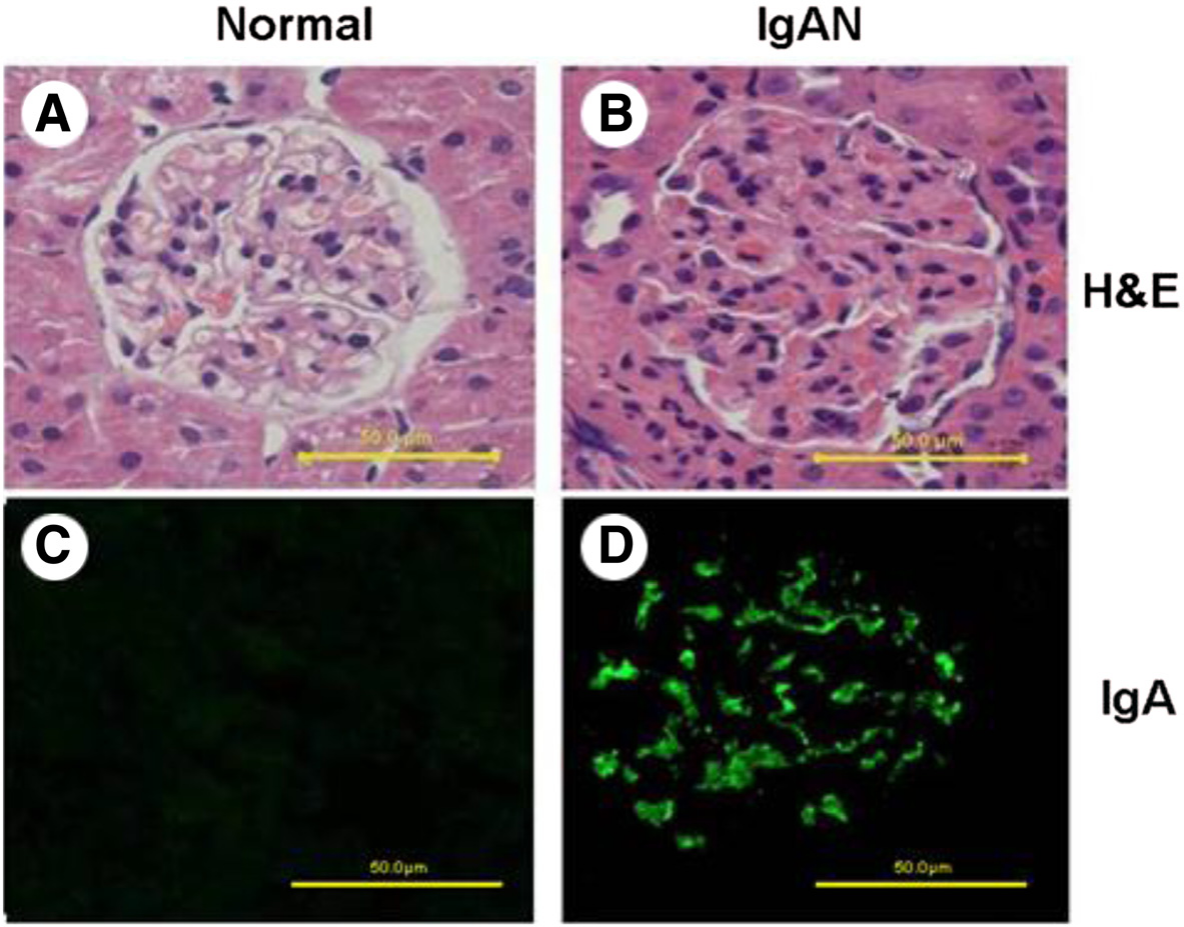
**Histopathology of kidney sections from control and IgAN rats.** The renal sections were stained by H&E staining **(A, B)** and IgA antibody followed by FITC 2^nd^ antibody **(C, D)**, respectively.

We examined the physical appearance and biochemical parameters in the rats. IgAN rats were found to show grey hair and slow-growing. 24-hour urine protein, Urinary red blood cell, BUN, Scr and IgA serum level were significantly elevated in IgAN rats. ALT, AST and GGT showed no significant change in IgAN rats after treatment compare to controls. (Table 2) Immunofluorescence studies showed that deposition of IgA was mainly seen at glomerular mesangial area, most of the staining in the IgAN model group was ++ to +++ (Figure 1).

**Table 2:** **Biochemical factors of normal and IgAN rats groups (**$\bar{x}$**± s)**

	**Normal Group**	**IgAN Group**
Weight (gram)	237.0 ± 9.7	204.5 ± 7.1*
Urinary red blood cell count (cells/μL)	4.82 ± 1.6	422.12 ± 49.65*
Urine Protein (mg/24 h)	9.94 ± 4.03	70.3 ± 33.2*
BUN (mmol/L)	6.57 ± 0.49	10.42 ± 1.32*
Scr (μmol /L)	26.8 ± 3.89	52.8 ± 6.76*
Serum IgA(μg/ml)	26.04 ± 1.12	28.99 ± 1.30*
ALT (u/L)	34.17 ± 3.87	40.17 ± 5.74
AST (u/L)	85.67 ± 11.65	90.5 ± 13.05
GGT (u/L)	28.0 ± 5.59	28.17 ± 5.53
ALB (g/L)	40.67 ± 4.72	38.0 ± 4.29

*P < 0.01, compare to normal group.

### Identify EPCs

**Figure 2:**
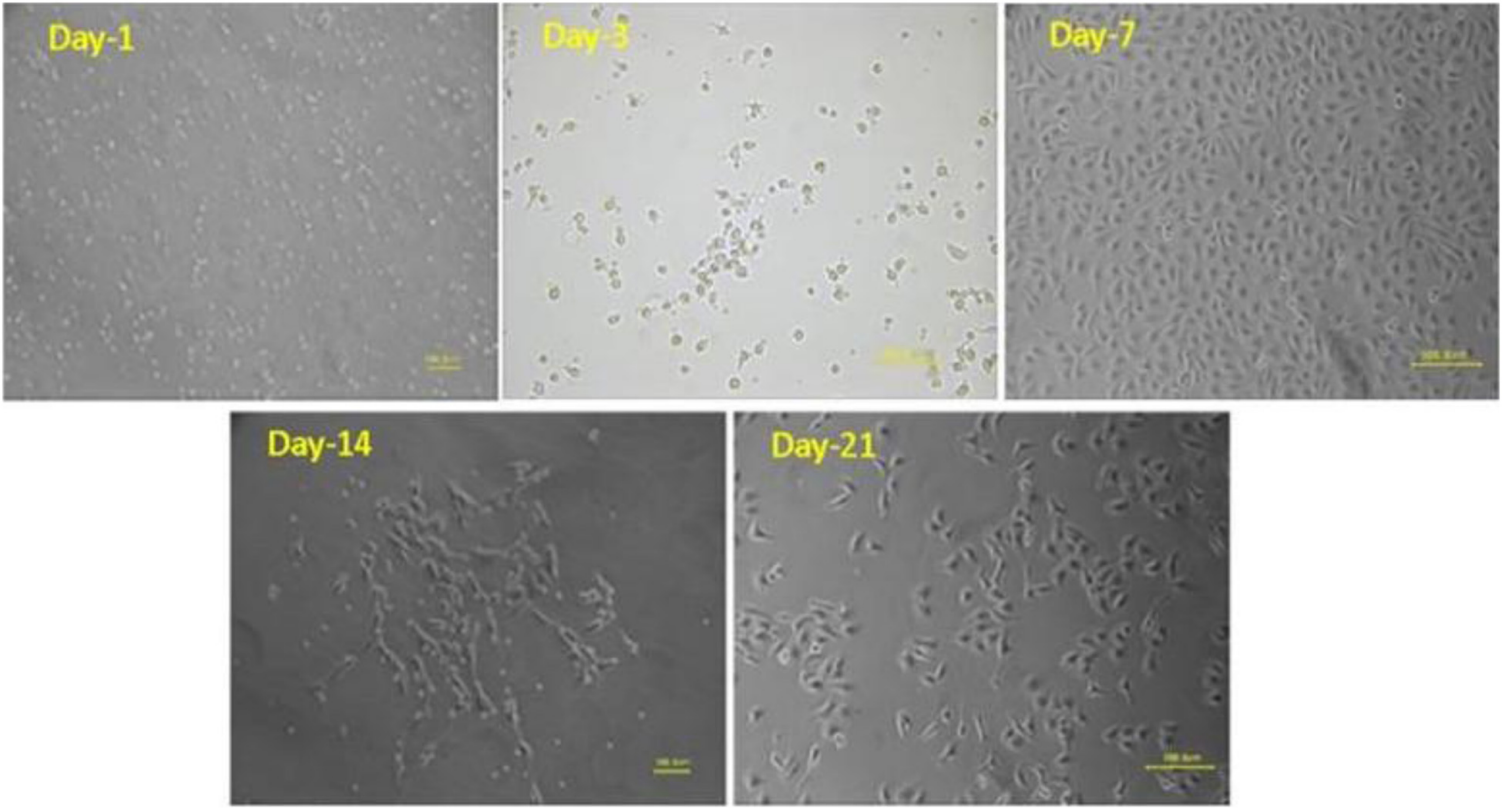
**The morphological changes of cultured endothelial progenitor cells (EPCs).** All images were taken under light microscope. Day-1, cells were small-round-shaped; Day-3, cells enlarged; Day-7, cells were short spindle sharped; Day-14, cells became slender-spindle-shaped and number of cells increased; Day-21, cells grew with a “cobblestone” appearance.

**Figure 3:**
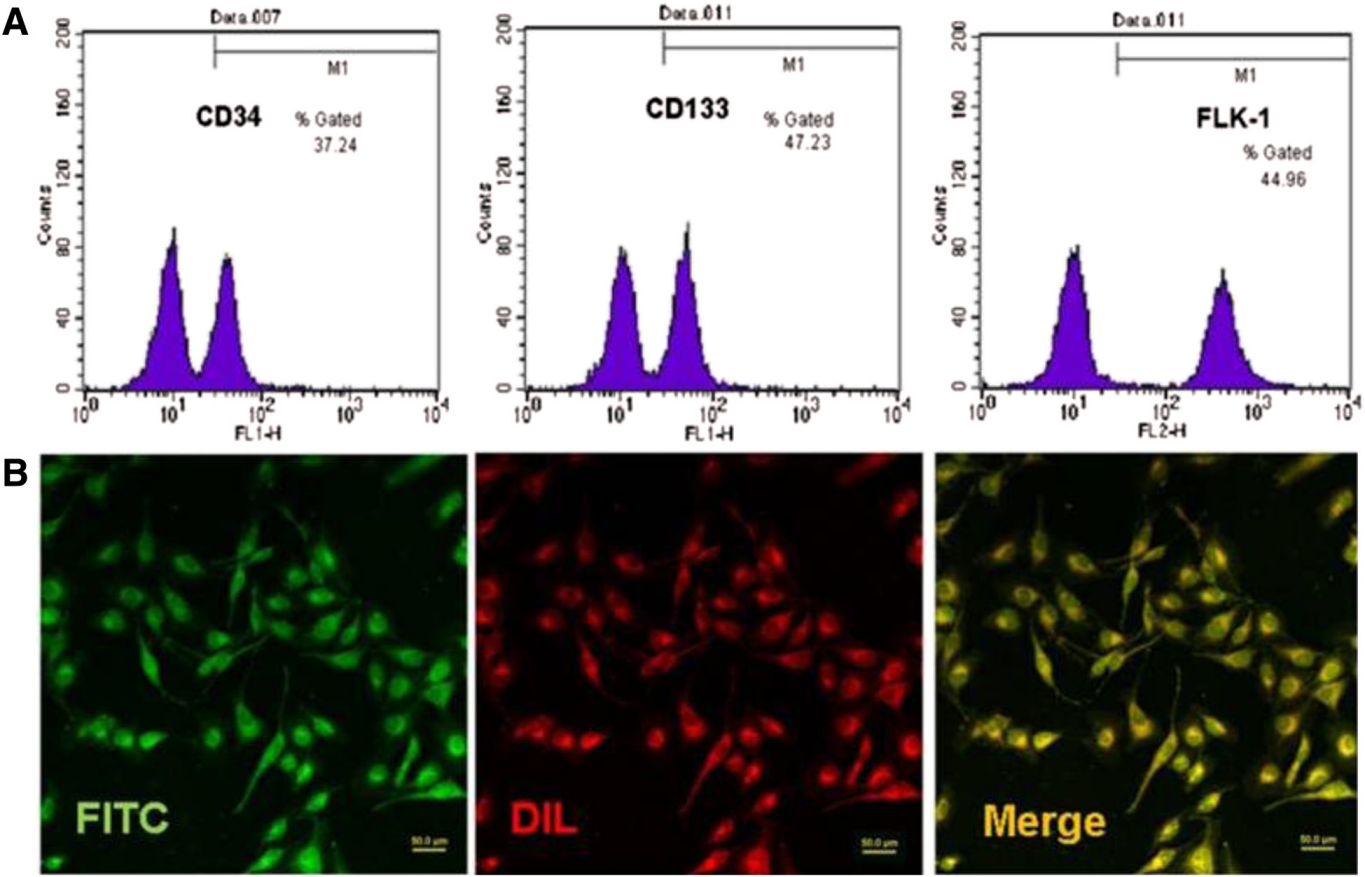
**Identification of EPCs.** The cells were collected and analyzed by flow cytometer for CD34, CD133 and FLK-1. The population of positive cells was indicated as percentage number in figures **(A)**. The cells were also fixed and dual stained with DiL-acLDL (red)/ FITC-UEA-1 (green). Merged images showed that most cells were dual-postive. Dual-postive cells were defined as EPCs **(B)**.

The freshly isolated EPCs were small-round shaped and suspended in culture medium. At day 2, cells started to attach to the dish wall, and cellular hypertrophy was observed at day 3, followed by becoming short spindle shaped. The cellular growth peak was reached after 7-day culture. At day 10–14, the cells became slender-spindle-shaped and started colony formation. At day 21, cell colony formation was completed with a “cobblestone” appearance, as shown in Figure 2. At day 14, EPCs were identified by CD34, CD133, FLK-1 using flow cytometer. DiL-acLDL (red)/FITC-UEA-1 (green) dual staining cells were observed under the laser scanning confocal microscopy. The cells with dual staining (shown as yellow fluorescence) were considered as differentiating EPCs (Figure 3).

### The expression of PKH26

**Figure 4:**
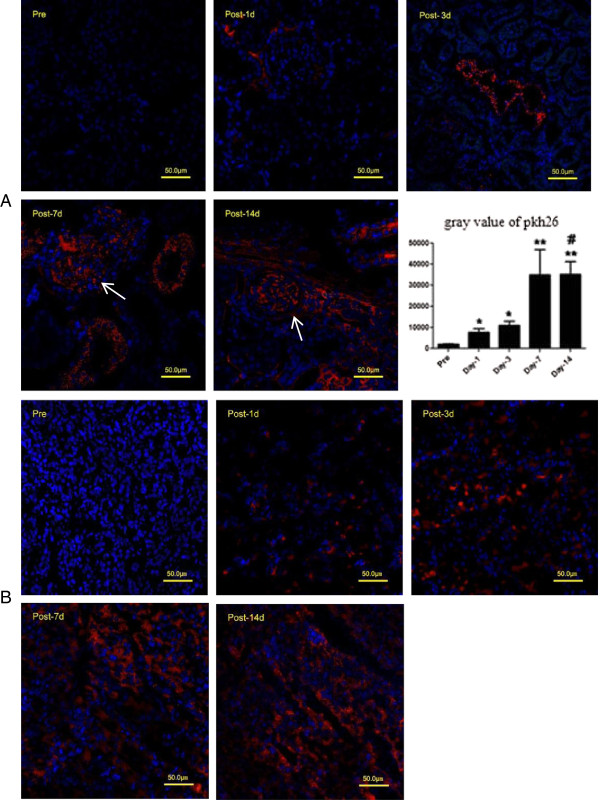
**Immunostaining of PKH26 on tissues from IgAN rats after EPCs transplantation.** In kidney tissue, the white arrows indicate the location of glomeruli. The intensity of PKH26 positive signal were quantified With MetaMorph software, and presented in column graph (bottom right). *P < 0.05, **P < 0.01, comparing to pre- transplantation, ^#^P < 0.05, comparing today-1 **(A)**. PKH26-positive signal in the liver tissue **(B)**.

Cryo-section of kidney and liver tissues stained with PKH26 and incubated with 4′, 6-diamidino-2-phenylindole (DAPI). PKH26 positive cells showed red fluorescence, nuclei were stained blue with DAPI under fluorescence microscopy. 1 day after transplantation, PKH26 positive cells were detected in the kidney of EPCs rats, mainly in the interstitial area. 3, 7, 14 days after transplantation, more and more PKH26 positive cells were discovered in glomerular and interstitial areas. The intensity of red fluorescence was gradually increased after transplantation. The difference between day-1 and day-14 after transplantation was significant. In addition to the kidney, PKH26-positive signal could also be detected in the liver tissue. The significance of this liver migration of EPCs was still waited to be investigated (Figure 4).

### Biochemical examination

24-hour urine protein, Urinary red blood cell, BUN, Scr and IgA serum level in IgAN group increased significantly compared to the ones in normal group. However, these parameters in EPCs group decreased compared to the ones in IgAN group. The differences between groups were statistically significant (p < 0.01, Table 3). After transplantation, 24-hour urine protein, Urinary red blood cell, BUN, Scr and IgA serum level gradually decreased. The differences between 14 days post-transplantation and 1 day prior to transplantation were statistically significant (p < 0.01, Table 4).

**Table 3:** **Biochemical parameters of Normal, IgAN and EPCs rats groups **($\bar{x}$**± s)**

	**Normal group**	**IgAN group**	**EPCs group**
Urine Protein (mg/24 h)	9.94 ± 4.03	77.7 ± 17.1	28.16 ± 8.04*^#^
Urinary red blood cell count(cells/μL)	5.17 ± 0.79	454.35 ± 72.3	323.68 ± 32.64**
BUN (mmol/L)	6.57 ± 0.45	11.02 ± 1.37	7.88 ± 0.36*^#^
Scr (μmol/L)	26.8 ± 3.53	55.4 ± 6.02	35.6 ± 5.91*^#^
Serum IgA(μg/ml)	26.37 ± 2.49	31.72 ± 3.73	27.73 ± 1.65**
ALT (u/L)	33.67 ± 4.76	33.33 ± 3.82	35.82 ± 5.71
AST (u/L)	86.0 ± 8.07	86.5 ± 6.65	88.1 ± 6.69
GGT (u/L)	25.5 ± 3.27	27.0 ± 2.19	27.17 ± 2.48
ALB (g/L)	37.67 ± 2.16	40.17 ± 2.32	39.23 ± 2.49

*P < 0.01, **P < 0.05, compare to IgAN group;

^#^*P* < 0.01, compare to Normal group.

**Table 4:** **Biochemical parameters in EPCs group after transplantation** ($\bar{x}$**± s)**

	**Day-0**	**Day-1**	**Day-3**	**Day-7**	**Day-14**
Urine Protein (mg/24 h)	70.3 ± 33.2	65.44 ± 16.9^#^	54.22 ± 16.2^#^	28.86 ± 8.56*	28.16 ± 8.0*
Urinary red blood cell count(cells/μL)	452.72 ± 45.8	444.5 ± 42.13^#^	432.88 ± 31.5^#^	406.9 ± 27.01^#^	323.68 ± 32.64*
BUN (mmol/L)	10.42 ± 1.18	10.06 ± 0.87^#^	9.6 ± 0.85^#^	8.76 ± 0.82*	7.88 ± 0.36*
Scr (μmol/L)	52.8 ± 6.05	52.6 ± 9.07^#^	45.2 ± 7.79^#^	38.4 ± 6.73*	35.6 ± 5.91**
Serum IgA(μg/ml)	30.37 ± 1.74	29.72 ± 2.24^#^	28.88 ± 1.82^#^	28.32 ± 1.87^#^	27.73 ± 1.65*

Comparing to Day-0: ^#^P > 0.05, *P < 0.05, **P < 0.01. Day-0 is the EPCs rats before receiving EPCs transplantation.

### Pathological observation of kidney

**Figure 5:**
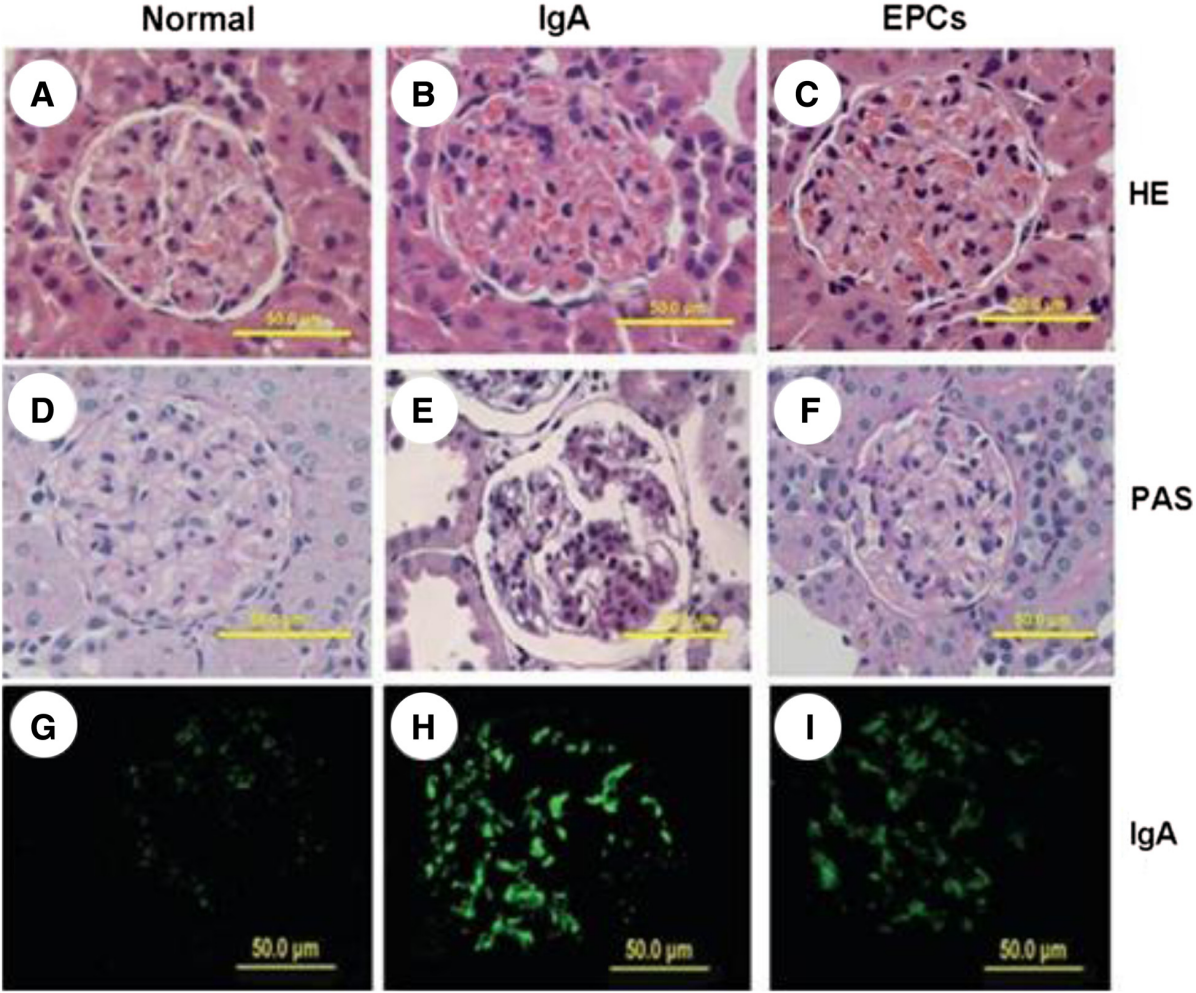
**Representative images of histological staining of renal sections.** The renal sections were stainied by H&E staining **(A, B, C)** , PAS staining **(D, E, F)** and Immunofluorescence staining for IgA **(G, H, I)**, respectively. Normal group, there was no significant histological abnormality after saline solution injection **(A, D, G)**, IgAN group after saline solution injection, the pathological changes were increased number of cells in the glomeruli, and moderate to severe hyperplasia of mesangial matrix and cells. There was significiant green fluorescence in the glomeruli. **(B, E, H)**. EPCs group after EPCs transplantation, hyperplasia of mesangial matrix and cells was decreased, green fluorescence in the glomeruli was weaken than IgAN group, **(C, F, I)**.

Morphology of glomerular, tubular, and renal interstitial remainined normal in normal group. While, in IgAN groups, the number of glomerular mesangial cells increased, mesangial matrix widened, the shape of renal tubules became irregular. In EPCs group, pathological changes of renal mesangial matrix and tubules were significantly alleviated (shown in Figure 5).

The PAS staining showed that in IgAN group, the ratio of ECM area and the corresponding glomerular area was significantly higher than the ratio in normal group (*p <* 0.01), suggesting the accumulation of renal glomerular ECM. However, in EPCs group, the deposition of renal glomerular ECM decreased significantly, shown by lower ratio of ECM area and the corresponding glomerular area (p < 0.01) (Figure 5 and Table 5).

**Table 5:** **The ratio of ECM to total glomerular area (ECM/TGA) and PTC density in normal/IgAN/EPCs group (**$\bar{x}$**± s)**

	**Normal group**	**IgAN group**	**EPCs group**
ECM/TGA(%)	0.44 ± 0.58	11.06 ± 1.4#	5.59 ± 1.94*^#^
PTC density (A)	44.75 ± 10.26	17.93 ± 4.15#	30.9 ± 3.37*^#^

*P < 0.01 compare to IgAN group; #P < 0.01compare to normal group.

### Immunofluorescence of IgA

Immunofluorescence staining of IgA showed slight IgA deposition in normal rats and the fluorescence intensity was scored as “–” to “+”. In contrast, IgAN rats had significant granule-like green fluorescence in the glomeruli which was scored as “++” to “++++”, these results indicated successful establishment of IgAN. EPC rats had significantly reduced green fluorescence in the glomeruli which was scored as “+” to “+++” compare to IgAN rats (Figure 5).

### Immunohistochemistry

**Figure 6:**
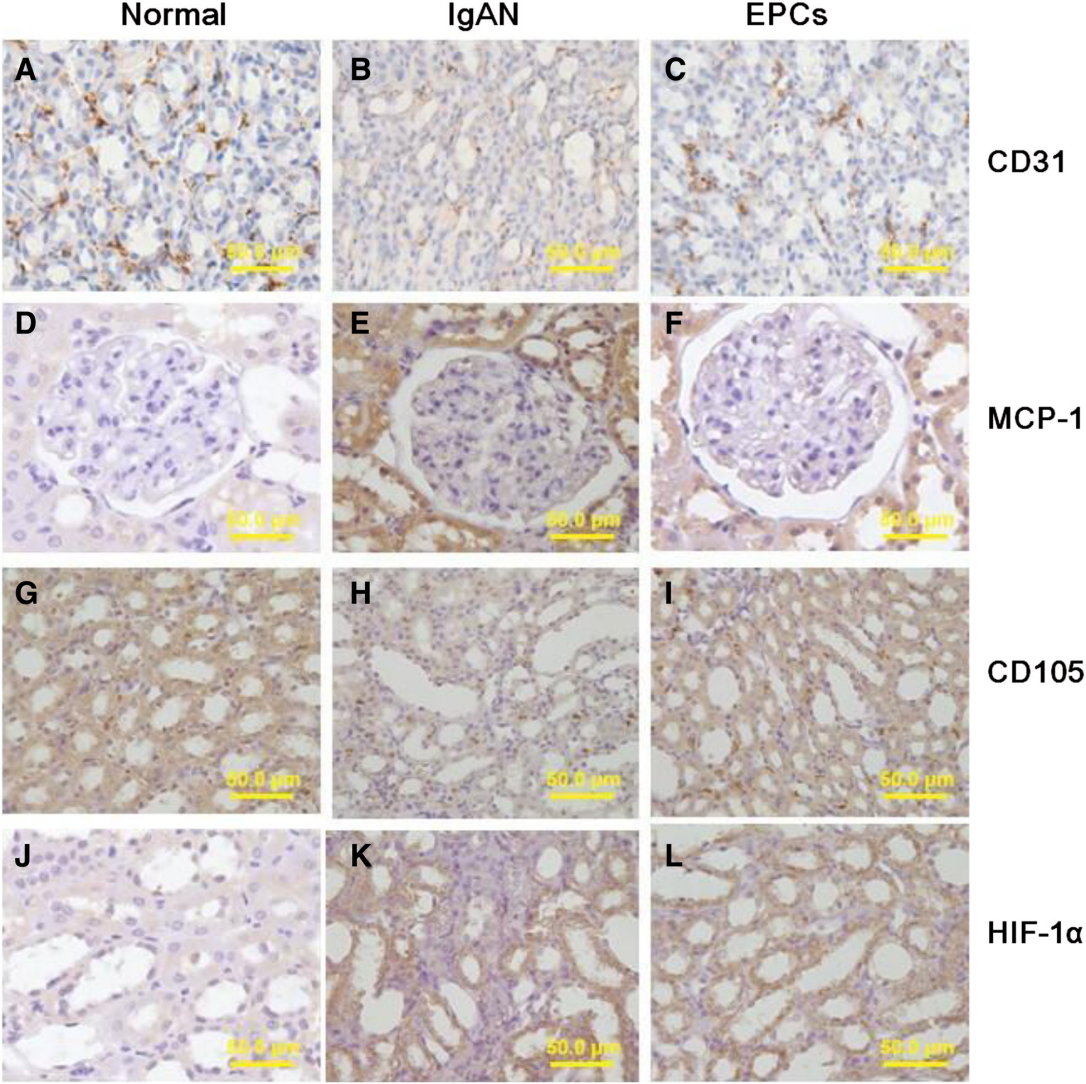
**Immunohistochemical detection of CD31 , MCP-1, CD105 and HIF-1a.** Normal group, CD31-positive endothelial cells were regularly distributed in renal interstitium **(A)**; The expression of CD31 was significantly decreased in IgAN group **(B)**; In EPCs group, the expression of CD31 was also decreased than the normal group,however, it was significantly increased compared to the one in IgAN group. **(C)**. In normal group, MCP-1 staining was barely detectable in renal tubular epithelial cells **(D)**; However, in IgAN group, the enhanced expression of MCP-1 was detected. **(E)**; MCP-1 expression gradually decreased in EPCs group **(F)**. Normal group, CD105 staining was weak. **(G)**; IgAN group, the expression of CD105 was significantly elevated. **(H)**; CD105 expression in EPCs group after transplantation. **(I)**. In normal group, minimal HIF-1α was detected in renal tubular epithelial. **(J)**; The expression of HIF-1α increased significantly in IgAN group, **(K)**; It gradually decreased in EPCs group **(L)**. Immunohistochemistry of CD31 **(A, B, C)**; Immunohistochemistry of MCP-1 **(D, E, F)**; Immunohistochemistry of CD105 **(G, H, I)**; Immunohistochemistry of HIF-1α **(J, K, L)**.

In normal group, CD31 was shown as brown fine deposits on the membrane of endothelial cells of PTC, which were distributed in renal interstitium among renal tubules; some was shown on the membrane of glomerular vascular endothelial cells. The expression of CD31 was significantly decreased in IgAN group as a comparison to the normal group, while the expression of CD31 in EPCs group was significantly increased compared to the one in IgAN group. Integrated optical density (IOD) value of CD31 was used to represent the density of peritubular capillary. The difference between groups was statistically significant (Figure 6, Tables 5 & 6).In normal group, MCP-1 staining was barely detectable in renal tubular epithelial cells. However, in IgAN group, the enhanced expression of MCP-1 was detected. After EPCs transplantation, MCP-1 expression gradually decreased at day-3, day-7 and day-14 (Figure 6).In normal group, CD105 staining was weak in stromal vascular endothelial cells and glomerular/interstitial cells. While in IgAN group, the expression of CD105 was significantly elevated prior to transplantation. CD105 expression in EPCs group diminished at day-3 after EPCs transplantation, while it increased at day 7 and day 14 (Figure 6).In normal group, minimal HIF-1α was detected in renal tubular epithelial. The expression of HIF-1α increased significantly in IgAN group, while it gradually decreased in EPCs group (Figure 6).

**Table 6:** **The ratio of ECM to total glomerular area (ECM/TGA) and PTC density in EPCs group after transplantation (**$\bar{x}$**±s )**

	**Day-0**	**Day-1**	**Day-3**	**Day-7**	**Day-14**
ECM/TGA(%)	11.06 ± 1.94	10.57 ± 1.8#	8.94 ± 1.71*	8.62 ± 1.13**	6.05 ± 1.49**
PTC density (A)	13.69 ± 3.54	14.96 ± 3.34#	19.26 ± 3.01*	24.62 ± 3.88**	30.9 ± 3.37**

Comparing to Day-0: ^#^P > 0.05,*P < 0.05,**P < 0.01. Day-0 is the EPCs rats before receiving EPCs transplantation.

### Protein and mRNA levels of MCP-1, HIF-1α and CD105

**Figure 7:**
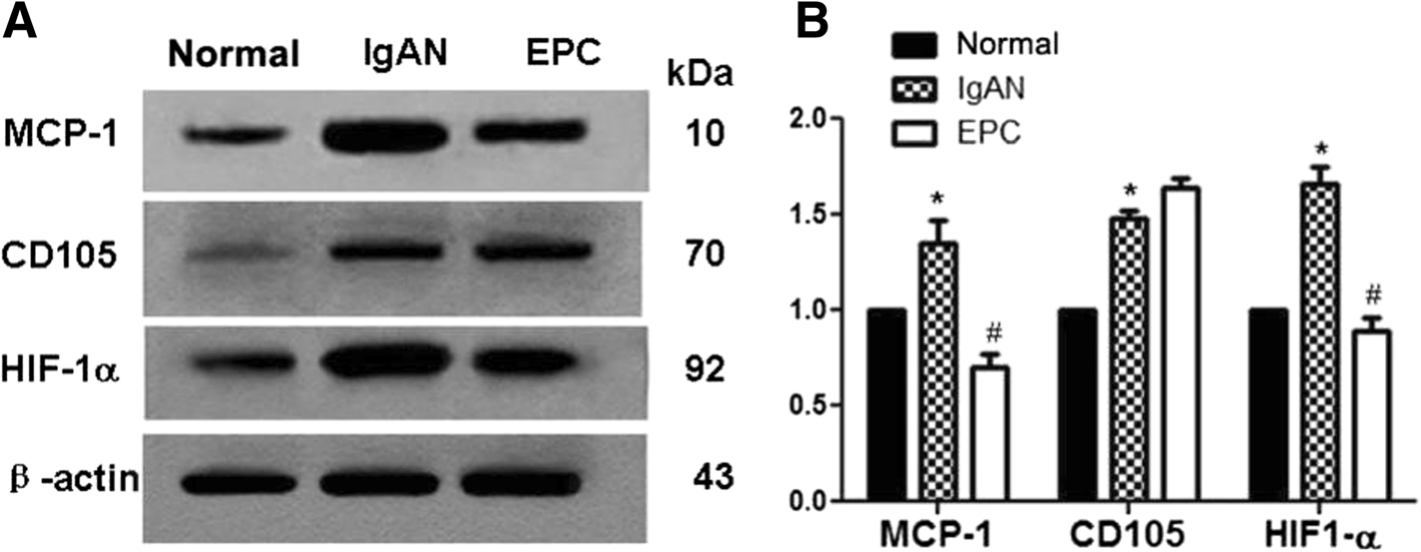
**Protein expression of MCP-1, CD105 and HIF-1α after transplantation.** Representative immunoblots **(A)** and quantitative densitometry analysis (**B**, from **5** experiments) demonstrating protein expression of MCP-1, CD105 and HIF-1α, in the renal cortex of rats from normal, IgAN and EPCs groups.*P < 0. 05 vs normal,^#^P < 0.05 vs IgAN.

**Figure 8:**
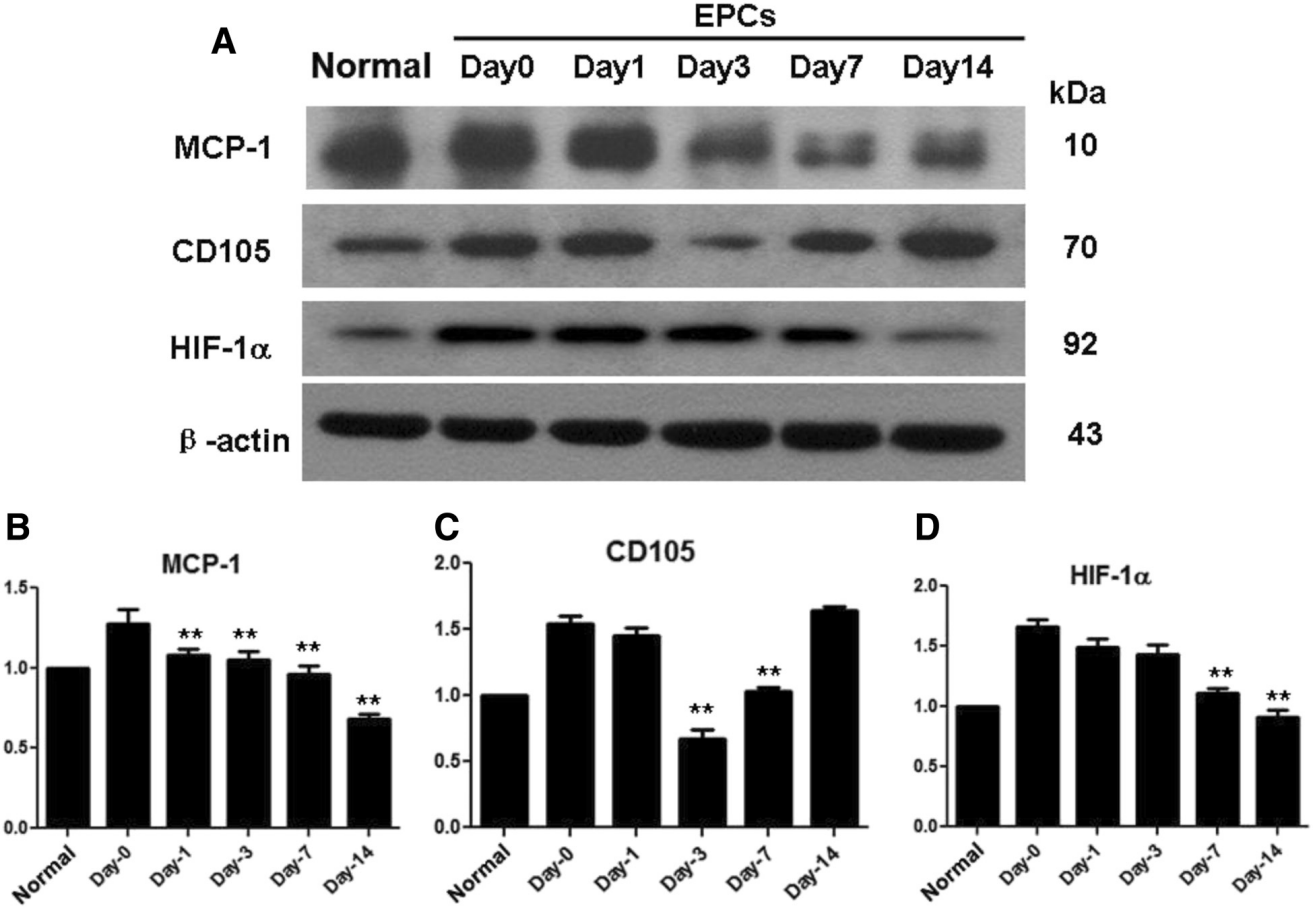
**Protein expression of MCP-1, CD105 and HIF-1α during transplantation.** Representative immunoblots **(A)** and quantitative densitometry analysis **(B, C, D)** for protein expression of MCP-1, CD105 and HIF-1α, in the renal cortex of rats from normal, day-0, day −1, day-3, day-7 and day14 EPCs rats after transplantation. *P < 0.05, **P < 0.01, comparing to the day-0 group.

**Figure 9:**
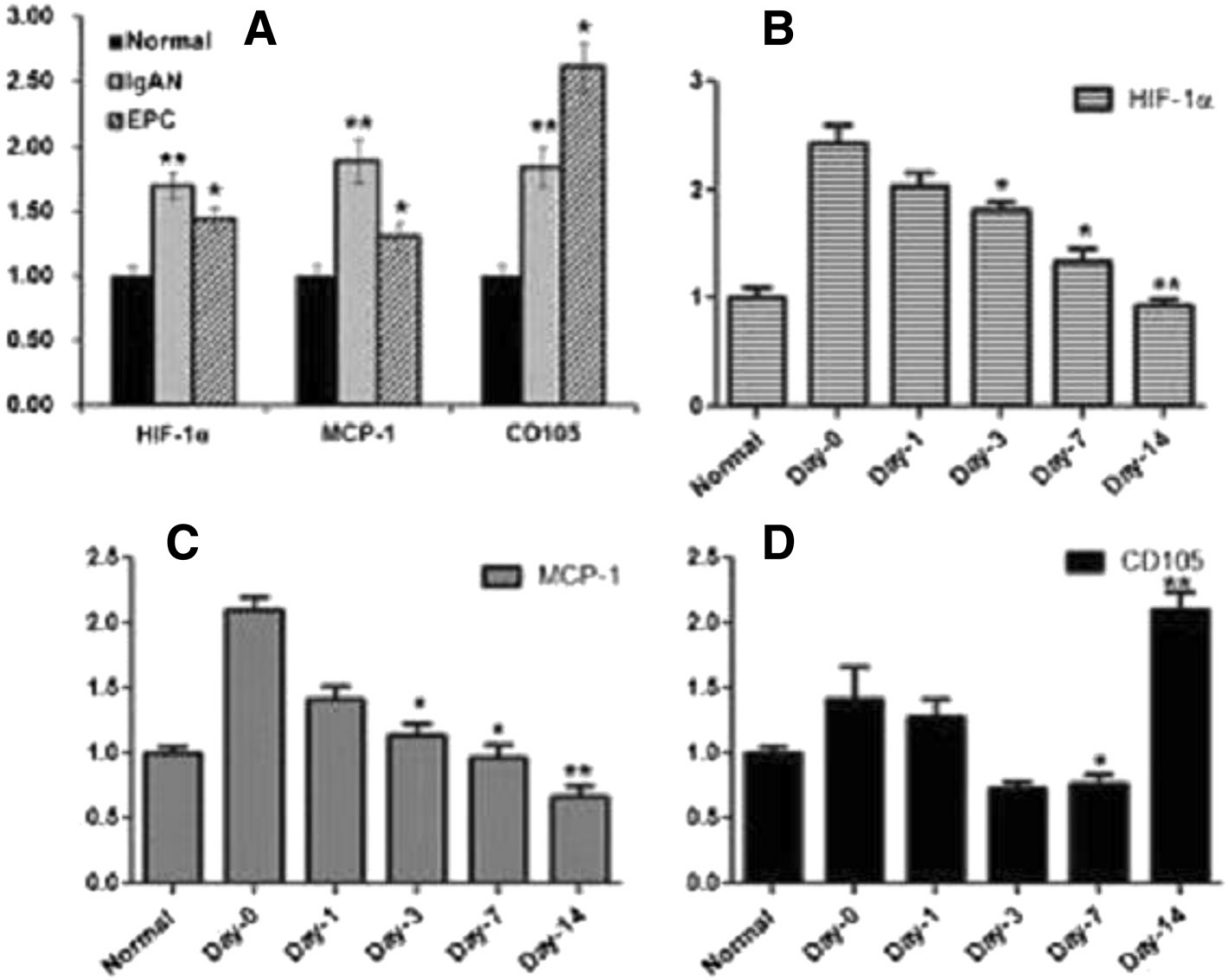
**mRNA level of MCP-1, CD105 and HIF-1α.** Real-time quantitative RT-PCR analysis of MCP-1, CD105 and HIF-1a mRNA after transplantation. **P < 0. 05 vs normal, *P < 0.05 vs IgAN. **(A)**. MCP-1, CD105 and HIF-1a mRNA from the renal cortex of rats isolated from normal, day-0, day −1, day-3, day-7 and day14 EPCs rats after transplantation. *P < 0.05,**P < 0.01, comparing to the day-0 group **(B, C, D)**.

To confirm the expression prolife of the molecules we observed in immuno- histochemistry studies, we measured the expression again by using quantitative western blotting or quantitative RT-PCR. Similarly, we found that normal group express relatively less amount of MCP-1, HIF-1α, which were significantly higher in IgAN groups. However, after EPC transplantation, MCP-1, HIF-1α expression was gradually decreased in 14 days. The protein expression of CD105 was decreased in the first 3 days after transplantation, while it increased in day-7 and day-14. mRNA of these molecules showed the similar results. mRNA levels of MCP-1 and HIF-1α were significantly increased in IgAN group, while the levels were gradually decreased in 14 days after transplantation. CD105’s mRNA first increased in the first 7 days after transplantation, but it increased and reached the peak at day-14 (Figures 7, 8 & 9).

## Discussion

Currently, IgAN is considered as a very severe immune disease, on which there is no proper and successful treatment. Our research aims to control or improve the pathogenesis of renal tissue in IgAN rat model, by PKH26-labeled EPCs transplantation. IgAN rat model was established by a combined injection of USA, LPS and CCL4. Hematuria and proteinuria were observed in the treated rats at 6 weeks post-injury and some of IgAN rats developed gross hematuria (data not shown). Obvious IgA deposition in the glomeruli of these animals could also been detected. These findings indicate a successful establishment of IgAN model in rats. One day after the transplantation, the PKH26-labeled cells started to express mainly in renal interstitial area. At the day 3, 7, 14, the number of PKH26-labeled cells in the renal interstitial and glomerular area gradually multiplied, indicating that intravenous injected BM-EPCs can be re-located to the damaged kidneys. It was observed that urinary red blood cell, 24- hour urine protein, BUN and Scr dramatically decreased as well as the improvement of renal pathological findings in EPCs group comparing to IgAN group. Other markers, such as HIF-1α and MCP-1 expression increased in IgAN group but decreased in EPCs group. While CD105 was an exception, the expression of CD105 sustained at a high level when EPCs were transplanted. All these data suggested that BM-EPCs transplantation significantly decreases the levels of inflammatory factors, and improved ischemic- induced renal tissue hypoxia.

The conventional immunosuppressive treatment, such as the corticosteroid and cytotoxic drugs, the application of ACEI and ARB have been introduced into the treatment of IgAN for many years, however, all these methods are not effective, especially for IgAN patient’s prognoses [13,14]. Among all the factors which affect IgAN prognoses, renal interstitial fibrosis is one of the most independent risk factors. PTC provides the major blood supply for renal tubule-interstitium; and much evidence has suggested that loss of PTC is mainly due to ischemic injury and fibrosis of renal tubule-interstitium. One major feature of PTC pathology is the decreased number of capillary endothelial cells. Therefore, repair or regeneration of endothelial cells and reducing microvascular loss will be the promising therapeutic treatment for IgAN. Endothelial progenitor cell-mediated therapeutic angiogenesis could be an effective way to control or improve a wide variety of pathological condition. In recent years, EPCs therapy has become a hot spot in the treatment of heart disease, stroke, peripheral arterial disease, rheumatism, cancer and other diseases [15-18]. In our laboratory, recent data showed that the cell number of bone marrow EPCs in IgAN rats was signficantly less than that of normal rats by 33.3%; the migration rate of these cells in IgAN rats was decreased by 10%; the proliferation rate of these cells was decreased by 18%, which were all statistically significant comparing to that of normal rats (unpublished data). To continue the studies, here we tried to treat IgAN rats with exogenous EPCs transplantation. The results showed that BM-EPCs can re-locate in the kidney of IgAN rats. The IgAN rats receiving EPCs have significantly improved kidney function, reduced expansion of glomerular extracellular matrix, attenuated IgA deposits in the glomeruli and increased density of peritubular capillary.

CD105, known as endoglin, is a type-I glycoprotein on cellular membrane. CD105 is predominately expressed in vascular endothelial cells and related tissues, while is absent in lymphatic endothelial cells [19]. Different from pan-vascular endothelial cell markers, CD105 is only strongly expressed in highly proliferating vascular endothelial cells, while weakly or not expressed in vascular endothelial cells in normal adult tissues. Other researchers have found that in the case of inflammation occurring on skin lesion, the up-regulation of CD105 promptly accelerated the infiltration of inflammatory cells and angiogenesis [20]. Our research also showed the higher expression of CD105 in the kidney tissue of IgAN rats, which might due to the elevated inflammatory responses in IgAN rats. CD105 expression decreased 3 days after EPCs transplantation, indicating the reduced inflammatory responses. At the day-7 and day-14 after transplantation, CD105 level increased gradually comparing to the day-3. We speculate this change might due to the process that relocated EPCs were transformed into actively proliferating vascular endothelial cells.

Another marker for vascular endothelial cells is CD31, which is highly expressed at the cellular junctions between vascular endothelial cells. Therefore, in this study, the number of CD31 positive cells was used to represent PTC density. In our research, CD31 positive staining was dramatically decreased in the kidney tissue of IgAN group comparing to that of normal group, indicating a decreased PTC. 7 and 14 days after transplantation, in EPCs group, the number of CD31 positive cells was increased, suggesting an increasing PTC density in EPCs group comparing to that in IgAN group.

Previous studies have shown that hypoxia is a major factor for cellular damage and angiogenic dysregulation. Hypoxia can induce the expression of certain genes and then affect cellular function, which is one of the important mechanisms for the initiation and development of renal interstitial fibrosis [21-23]. The declining of PTC density often leads to tubule-interstitial in a hypoxic state. Hypoxia-inducible factor 1-α (HIF-1α) is a transcription factor in cells growing at low oxygen concentration, which plays an essential role in cellular and systematic responses to hypoxia [24,25]. Therefore, HIF-1α is often used as a sensitive indicator for hypoxia. Our study showed that HIF-1α expression increased in the kidney tissues of IgAN rats, while decreased in EPCs rats (*p <* 0.05), indicating that EPCs transplantation could improve hypoxic condition of tubule-interstitial. This result is consistent with the observation that PTC density was increased in EPCs rats.

MCP-1 is an inflammatory chemokine. Local overexpression of MCP-1 at vessel wall induces infiltration and aggregation of mono-nuclear cells, and cause formation of atherosclerotic lesion [26]. It also promotes the expression of important cytokines which cause fibrosis, such as IL-6, TGF-β, etc. In kidney, overexpression of MCP-1 results in the accumulation of extracellular matrix in glomerular and tubules, causing glomerulosclerosis and renal interstitial fibrosis, eventually leading to renal failure [27]. Only trace expression of MCP-1 was detected in renal mesangial cells, renal tubular epithelial cells, vascular endothelial cells and other cells under normal circumstances. However, MCP-1 is induced and highly expressed under many other conditions, such as hypoxia, immune responses, viral infection, injury, hemodynamic changes, etc. [28,29], which is in agreement with our observation. We found that MCP-1 expression was enhanced in the kidney of IgAN group comparing to the normal group, while it declined after EPCs transplantation. The differences between day-7/14 and day-prior-to transplantation were statistically significant.

In IgAN pathogenesis, ECM expansion or mesangial proliferation is often associated with serum elevation of IgA-IgG2a IC and enhanced deposition of IgA, as well as cytokine production such as IL-6 and TGF-β. Therefore, in this study the ratio of the ECM area to glomerular cross-sectional area was determined to represent the degree of nephropathy in IgAN experimental rats. We found that this ratio was significantly decreased in EPCs transplanted rats, comparing to IgAN group, suggesting that EPCs transplantation can cause a decline in the deposition of ECM in mesangial area. It’s been known that the increased synthesis and declined degradation of ECM are the important factors causing the development of glomerulosclerosis [30-32]. Therefore, in theory, EPC transplantation may further delay the progress of glomerular sclerosis, which may need further investigation.

In summary, our findings suggested that BM-EPCs were re-located to the damaged kidneys and improve the pathogenesis of renal tissue in IgAN rat. It seemed provided a practically effective way for treating IgAN patients.

## Conclusion

EPCs transplantation in IgAN rats promotes angiogenesis, resulting in an increased PTC density. It also significantly down-regulates HIF-1α, MCP-1 expression, relieves tissue ischemia, improves hypoxia and inflammatory infiltration. In transplanted IgAN rats, the expansion of extracellular matrix in glomerular was reduced; renal pathogenesis progression was slowed, and renal function was improved. All these data establish the experimental fundament for the new therapies of IgAN.

## Competing interest

The authors declare that they have no competing interests.

## Authors’ contribution

WG (designed and finalized most of the work), JMF (conceived of the study and revised the article), LY (participated in the design of the study), LS (drafted the manuscript and performed the statistical analysis), GQZ (participating in the work of cell culture), All authors read and approved the final manuscript.

## Pre-publication history

The pre-publication history for this paper can be accessed here:

http://www.biomedcentral.com/1471-2369/15/110/prepub

## Supplementary Material

Additional file 1 ARRIVE checklist. Click here for file
